# Assessing susceptibility for polycyclic aromatic hydrocarbon toxicity in an *in vitro* 3D respiratory model for asthma

**DOI:** 10.3389/ftox.2024.1287863

**Published:** 2024-04-19

**Authors:** Reese M. Valdez, Brianna N. Rivera, Yvonne Chang, Jamie M. Pennington, Kay A. Fischer, Christiane V. Löhr, Susan C. Tilton

**Affiliations:** ^1^ Environmental and Molecular Toxicology Department, Oregon State University, Corvallis, OR, United States; ^2^ Superfund Research Program, Oregon State University, Corvallis, OR, United States; ^3^ Oregon Veterinary Diagnostic Laboratory, College of Veterinary Medicine, Oregon State University, Corvallis, OR, United States; ^4^ Department of Biomedical Sciences, Oregon State University, Corvallis, OR, United States

**Keywords:** polycyclic aromatic hydrocarbons, benzo[a]pyrene, lung cells, asthma, 3D *in vitro* model

## Abstract

There is increased emphasis on understanding cumulative risk from the combined effects of chemical and non-chemical stressors as it relates to public health. Recent animal studies have identified pulmonary inflammation as a possible modifier and risk factor for chemical toxicity in the lung after exposure to inhaled pollutants; however, little is known about specific interactions and potential mechanisms of action. In this study, primary human bronchial epithelial cells (HBEC) cultured in 3D at the air-liquid interface (ALI) are utilized as a physiologically relevant model to evaluate the effects of inflammation on toxicity of polycyclic aromatic hydrocarbons (PAHs), a class of contaminants generated from incomplete combustion of fossil fuels. Normal HBEC were differentiated in the presence of IL-13 for 14 days to induce a profibrotic phenotype similar to asthma. Fully differentiated normal and IL-13 phenotype HBEC were treated with benzo[a]pyrene (BAP; 1–40 μg/mL) or 1% DMSO/PBS vehicle at the ALI for 48 h. Cells were evaluated for cytotoxicity, barrier integrity, and transcriptional biomarkers of chemical metabolism and inflammation by quantitative PCR. Cells with the IL-13 phenotype treated with BAP result in significantly (*p* < 0.05) decreased barrier integrity, less than 50% compared to normal cells. The effect of BAP in the IL-13 phenotype was more apparent when evaluating transcriptional biomarkers of barrier integrity in addition to markers of mucus production, goblet cell hyperplasia, type 2 asthmatic inflammation and chemical metabolism, which all resulted in dose-dependent changes (*p* < 0.05) in the presence of BAP. Additionally, RNA sequencing data showed that the HBEC with the IL-13 phenotype may have increased potential for uncontrolled proliferation and decreased capacity for immune response after BAP exposure compared to normal phenotype HBEC. These data are the first to evaluate the role of combined environmental factors associated with inflammation from pre-existing disease and PAH exposure on pulmonary toxicity in a physiologically relevant human *in vitro* model.

## 1 Introduction

There is increased emphasis on understanding cumulative risk from the combined effects of chemical and non-chemical stressors as it relates to public health. In particular, cumulative risk assessments have not adequately addressed vulnerable and susceptible populations impacted by combined stressors due to lack of knowledge about how nonchemical stressors contribute to risk (Sexton, 2012; Rider et al., 2014). Recent animal and epidemiological studies have identified pulmonary inflammation as a contributing stressor and risk factor for chemical toxicity in the lung after exposure to inhaled pollutants, such as polycyclic aromatic hydrocarbons (PAHs); however, the interaction between PAHs and inflammatory airway diseases, such as asthma, is not well understood (Arlt et al., 2015). While several studies have shown that exposure to PAHs and ambient air pollution is associated with the development and exacerbation of asthma, there is a large knowledge gap in understanding how individuals with diseases like asthma may respond differently to chemical insult compared to normal or healthy individuals (Ren et al., 2014; Liu et al., 2016; Mei et al., 2018; Låg et al., 2020).

Asthma is one of the most common chronic noncommunicable disease impacting 16% of children and adults globally (Masoli et al., 2004). According to the World Health Organization (WHO), asthma affected about 262 million people and caused 455,000 deaths globally in 2019 (World Health Organization, 2022). Asthma is generally associated with airway hyperresponsiveness, inflammation, and mucus hypersecretion. The overall mechanism of asthma is not fully understood, but airway inflammation is believed to be central to disease pathophysiology (Dharmage et al., 2019). The inflammatory response in asthma contributes to remodeling of the airway. Airway hyperresponsiveness and remodeling can result in increased susceptibility to a number of environmental factors, including chemical insult, due to altered biological response and insufficient repair capacity (Holgate, 2012). Asthma has more recently been recognized as a heterogenous disease with multiple phenotypes and characteristics. One of the more common types of asthma is Th2 allergic asthma as 80% of children and 60% adult asthma cases are associated with this form (Marone et al., 2019; León and Ballesteros-Tato, 2021). Th2 allergic asthma is associated with many markers of type 2 inflammation, notably high levels of interleukin-13 (IL-13). IL-13 is a cytokine that can be produced by several different immune cells when stimulated by other cytokines released from epithelial cells. IL-13 has been shown to increase the proportion of secretory cells via goblet cell metaplasia and disrupting ciliated cell differentiation (Laoukili et al., 2001). The combination of increased goblet cells and decreased ciliated cells causes mucocilliary dysfunction, a hallmark of type 2 inflammation present in asthma and similar diseases (Maspero et al., 2022).

Previous studies have been successful in generating an asthma-like phenotype *in vitro* by differentiating airway epithelial cells cultured at the air-liquid interface (ALI) in the presence of IL-13 (Laoukili et al., 2001; Mertens et al., 2017; Jackson et al., 2020). Organotypic culture of human bronchial epithelial cells (HBEC) differentiated at the ALI to generate a 3D pseudostratified structure has been proposed as a viable alternative to animal models due the ability to recapitulate structure and multicellular function of the airway *in vivo* (Rayner et al., 2019). Over the past decade, there has been increasing interest in the development of advanced *in vitro* and tissue models to replace or complement animal studies for toxicity testing. In particular, models for 3D cells, tissues and multi-organ on chip technologies have been developed for predicting inhalation toxicity and airway disease in humans (Bedford et al., 2022). Primary cells cultured at the ALI have been found to mimic transcriptional response more similar to the bronchial epithelium in humans compared to traditional cell culture or use of transformed cell lines (Pezzulo et al., 2011). The three-dimensional nature of the cell culture model allows the cultures to resemble *in vivo* structures, ultimately allowing for responses comparable to an *in vivo* response. The bronchial epithelium functions as a physical barrier against environmental insults, such as particles, and utilizes airway mucus secretion and ciliated cell clearance together to create the mucociliary escalator to strengthen the barrier (Davies, 2014; Sözener et al., 2020). The epithelial cells that make up the barrier are also connected through tight junctions and apical junctions which contribute to regulating paracellular space passage and cell-cell adhesion (Sözener et al., 2020).

The class of contaminants known as polycyclic aromatic hydrocarbons (PAHs) are ubiquitous in the environment formed during incomplete combustion of natural sources, such as forest fire, or anthropogenic sources, such as cigarette smoke (Abdel-Shafy and Mansour, 2016). As such, exposure by inhalation is considered one of the primary ways humans are exposed (Ramesh et al., 2012; Abdel-Shafy and Mansour, 2016). Chemicals from this class represent some of the top chemicals of concern from priority pollutant sites having been identified by the United States Environmental Protection Agency (EPA) due to their potential toxicity and prevalence and persistence in the environment (Hussar et al., 2012). In addition, 15 PAHs have been identified as having at least some possible carcinogenic effect in humans by the International Agency for Research on Cancer (IARC) (Jameson, 2019). Benzo[a]pyrene (BAP) is a PAH that has been identified both as priority pollutant by the EPA and classified as carcinogenic to humans by IARC. BAP has also been associated with various types of cancer, including lung cancer, when metabolized (Abdel-Shafy and Mansour, 2016). While the mechanistic understanding of BAP and PAHs, in general, is largely based on knowledge of carcinogenic processes, these processes may also enhance non-malignant respiratory outcomes (Låg et al., 2020). BAP, as well as other PAHs, has been associated with a variety of health outcomes mediated by the aryl hydrocarbon receptor (AhR) including, but not limited to, immune modulatory effects, oxidative stress and disruption to the lung epithelial barrier suggesting that the overall respiratory effects of PAHs extends beyond carcinogenesis (Låg et al., 2020).

Both BAP and asthma are thought to impact the integrity of the barrier created by the bronchial epithelium. The specific mechanism that causes decreased barrier function after exposure is not fully understood; however, it has previously been shown that PAH exposure in the 3D HBEC model caused a functional decrease in barrier integrity as well as decreased expression of barrier function genes (Chang et al., 2019). One proposed mechanism of PAH-induced loss of barrier integrity is through the metabolism of PAHs. As PAHs are metabolized by the cytochrome P450 enzymes CYP1A1 and CYP1B1, reactive oxygen species and reactive electrophilic metabolites may be formed leading to inflammation and inhibition of barrier proteins (Overgaard et al., 2011; Låg et al., 2020). Similarly, inflammation and oxidative stress are central in asthma which both contribute to barrier dysfunction (Marone et al., 2019; Låg et al., 2020; Maspero et al., 2022). In this study, we evaluated the combined effects of PAH toxicity and IL-13-induced asthmatic inflammation in primary HBEC differentiated at the air-liquid interface to evaluate whether the IL-13 phenotype has altered susceptibility to BAP toxicity compared to cells with the normal phenotype. In addition, these studies explore the biological processes associated with Th2 airway remodeling *in vitro* in the presence and absence of chemical insult by BAP.

## 2 Materials and methods

### 2.1 Chemicals and reagents

Cell culture media was provided by STEMCELL Technologies (Vancouver, CA). Benzo[a]pyrene (BAP) (CAS# 50-32-8) was purchased from MRIGlobal (Kansas City, MO). Interleukin-13 (IL-13) was purchased from R&D Systems (Minneapolis, MN). Ultra pure dimethyl sulfoxide (DMSO) (CAS# 67-68-5) was purchased from VWR Chemicals (Solon, OH). Gibco™ Dulbecco’s phosphate-buffered saline without calcium and magnesium ions (DPBS), qPCR primers, and Pierce™ LDH Cytotoxicity Assay Kit were from Thermo Fisher Scientific (Waltham, MA). 2X SsoAdvanced™ Universal SYBR®Green Supermix and iScript™ Reverse Transcription Supermix for RT-qPCR were purchased from BioRad Laboratories, Inc. (Hercules, CA.)

### 2.2 Tissue culture and treatments

Primary HBEC (Lot #464078, Lonza, Morristown, NJ) at passage 2 were expanded until 80%–90% confluent, harvested by trypsinization and the cells were transferred to transwell inserts (Corning, 3,470, Kennebunk, ME) for differentiation at the air-liquid interface in 24-well plates each containing 500 uL of culture medium utilizing the PnuemaCult™ ALI medium (STEMCELL Technologies, Vancouver, CA) prepared per manufacturer’s instructions. Cells were cultured at 37°C, 5% CO2 with media changes every 48–65 h. On day 10, 10 ng/mL IL-13 was included in the media of cells to induce an asthmatic phenotype. After day 14, cells were washed with DPBS (pH 7.0–7.3) in triplicate once every 7 days in order to remove excess mucus from the apical surface. On day 25, inserts were washed with DPBS and then exposed on the apical surface for 48 h to either 25 uL vehicle control (1% DMSO in DPBS) or BAP (1–40 μg/mL) in 1% DMSO in DPBS. The tissues were removed from the insert, stored in QIAzol^®^ lysis reagent for RNA isolation, and frozen at −80°C. Basal media was collected in sterile tubes and stored at −80°C for evaluation of cytotoxicity. Samples for RNAseq analysis with 40 μg/mL BAP were collected in a separate experiment from the full dose-response study for qPCR (1–40 μg/mL) with endpoints of LDH and TEER measured for each. In addition, samples evaluated for histology were collected across all experiments.

### 2.3 Histology

HBEC cultures were washed briefly with phosphate-buffered saline (PBS, pH 7.4), excised from the culture inserts with a surgical blade, fixed in 10% neutral buffered formalin for 48 h prior to processing and embedding in paraffin. For staining and immunohistochemistry, 4 to 5-μm-thick sections were cut, mounted on slides, and deparaffinized by processing through a series of xylene and ethanol solutions. Sections were stained with hematoxylin and eosin (H&E) and mucus-producing goblet cells identified by periodic acid Schiff’s (PAS) staining. Sections of cell culture membranes also were stained for p63 and Ki-67 by immunohistochemistry (IHC). For IHC staining, paraffin sections were high-temperature antigen retrieved with Target retrieval solution (Agilent-Dako) (s1699). Endogenous peroxidase activity was blocked by immersing slides in Tris Buffered Saline with Tween (TBST Biocare Medical) containing 3% hydrogen peroxide for 10 min. The following primary antihuman antibodies were applied for 30 min at room temperature: rabbit polyclonal antiserum against human p63 (1:1,000; PA5-36069; ThermoFisher, Rockford, IL) and Ki-67 (1:1,000; PA5-16785; ThermoFisher, Rockford, IL). MaxPoly-One Polymer HRP Rabbit Detection solution (MaxVision Biosciences, Bothell, WA) was applied for 7 min at room temperature and Nova Red (SK-4800; Vector Labs, Burlingame, CA) as chromagen was used with Gill hematoxylin as counterstain. Serial sections of formalin-fixed paraffin-embedded cell culture membranes incubated with Dako Universal negative serum served as negative controls. Images were evaluated for relative differences between phenotypes by inspection of features and number or intensity of stained cells.

### 2.4 Transepithelial electrical resistance (TEER)

Transepithelial electrical resistance (TEER) was measured using an epithelial volt-ohmmeter (EVOM2, World Precision Instruments, Sarasota, FL), following the methods previously described (Chang et al., 2019). Briefly, the volt-ohmmeter was calibrated using a test electrode prior to the measurements. At time zero and 48 h after treatment with BAP or vehicle, DPBS was added to the apical chamber and the probe was added to both apical and basal chambers and resistance was measured (ohms) for each insert. In order to correct for the background resistance, an empty culture insert with DPBS in the apical and basal chambers was also measured. Four cultures were used for each treatment concentration and time point. Percent TEER of control was calculated by subtracting background from all values, then calculating the difference between each treatment group compared to DPBS vehicle control in normal phenotype cells.

### 2.5 Cytotoxicity

Lactate dehydrogenase (LDH) leakage was measured following methods previously described (Chang et al., 2019). Briefly, LDH leakage was measured in media 48 h after treatment with BAP or DPBS using a Pierce™ LDH Cytotoxicity Assay Kit (ThermoFisher), following the manufacturer’s instructions. Briefly, basal medium samples (25 µL) were aliquoted into the wells of a 96-well plate. LDH reaction reagent (25 µL) was added to each sample and incubated at room temperature for 30 min while protected from light. Finally, stop solution (25 µL) was added to each well and mixed. LDH activity was determined by subtracting absorbance at 680 nm (background) from absorbance at 490 nm using a Synergy HTX plate reader (BioTek, Winooski, VT).

### 2.6 RNA isolation and mRNA-Seq

Total RNA was isolated from HBEC (n = 15) using RNeasy Mini Kits (Qiagen) following the manufacturer’s protocol and was quantified on a Synergy HTX plate reader equipped with a Take3 module (BioTek, Winooski, VT). Samples exposed to DPBS and 40 μg/mL BAP in both the normal and IL-13 induced asthmatic phenotype (n = 4 per treatment) were selected for sequencing. RNA quantity was evaluated based on 280/260 ratio. RNA integrity (RIN) was assessed using an Agilent Bioanalyzer (Agilent, Santa Clara, CA) with samples having values ≥8.0 used for library construction and sequencing on a BGISEQ-500 platform at the Beijing Genomic Institute (BGI) Americas (www.bgi.com) with a sequencing length of 100 bp paired-end. For library construction, mRNA was poly-A selected and libraries were prepared as previously described (Dasgupta et al., 2022). Following mRNA sequencing, high quality reads were annotated and mapped to the human reference genome GRCh38. p12 using HISAT and differential expression was estimated using DESeq2 platform. The lists of differentially expressed genes (q < 1 × 10^−7^, |log2FC|>0.58) for each treatment were separated into upregulated and downregulated groups by treatment. Venn diagrams for upregulated and downregulated genes were generated in JMP Pro (Version 16.0.0; SAS, Cary, NC). The same upregulated and downregulated lists for each treatment were used in MetaCore (GeneGo, Thomson Reuters, Carlsbad, CA) for pathway enrichment analysis. Z-scores were used to compare expression in the normal and IL-13 induced asthmatic phenotypes after exposure to BAP. Significant biological process networks and enriched transcription factor analysis based on z-score transformations were determined with MetaCore (GeneGo) Heatmap visualizations were generated in BioVinci (BioTuring, San Diego, CA) and Multi-Experiment Viewer (Saeed et al., 2003). Network interaction visualizations were generated in Cytoscape (Version 3.9.1) utilizing the Compound Spring Embedder layout (Shannon et al., 2003).

### 2.7 Quantitative PCR (qPCR)

cDNA was synthesized from total RNA (section 2.6) using an iScript™ Reverse Transcription Supermix for RT-qPCR kit per manufacturer’s instructions. Reactions were diluted with nuclease-free water and stored in 20 µL aliquots at −80°C until used for qPCR. A BioRad Laboratories, Inc. (Hercules, CA) CFX96 Touch Real-Time PCR Detection System was used for running 10 µL qPCR reactions to survey key gene targets. Each reaction contained 2 µL of cDNA template, 150 nM of each primer, 5 µL of SsoAdvanced Universal SYBER Green Supermix, and nuclease-free water. A list of primer sequences is reported in Supplementary Table S1. Primer efficiency exceeded 85% for all primer sets. The thermocycler was programmed for 1 cycle 95°C for 1 min initial denaturing, 40 cycles 95°C for 15 s denaturing, 60°C for 30 s annealing/elongation, and a melt curve 65°C–95°C/0.5° per 5 s for validating single product amplification. The relative expression differences among treatments were calculated using the ΔΔCt comparative method and normalized to housekeeping gene peptidylprolyl isomerase A (*PPIA*). *PPIA* was determined to remain stable (*p* > 0.05) across conditions used in this study, including BAP treatment and IL-13 phenotype, as previously reported (Chang et al., 2019).

### 2.8 Statistical analysis

TEER, LDH, and qPCR were evaluated by removing any outliers identified by Grubbs’ test for outliers. Significant responses (*p* < 0.05) from the cleaned data sets were analyzed by one-way ANOVA with Fisher’s Least Significant Difference test. The potential for interactions between chemical treatment and phenotype was assessed by two-way ANOVA.

## 3 Results

### 3.1 Evaluation of HBEC differentiation cultured at ALI in presence of IL-13

**FIGURE 1:**
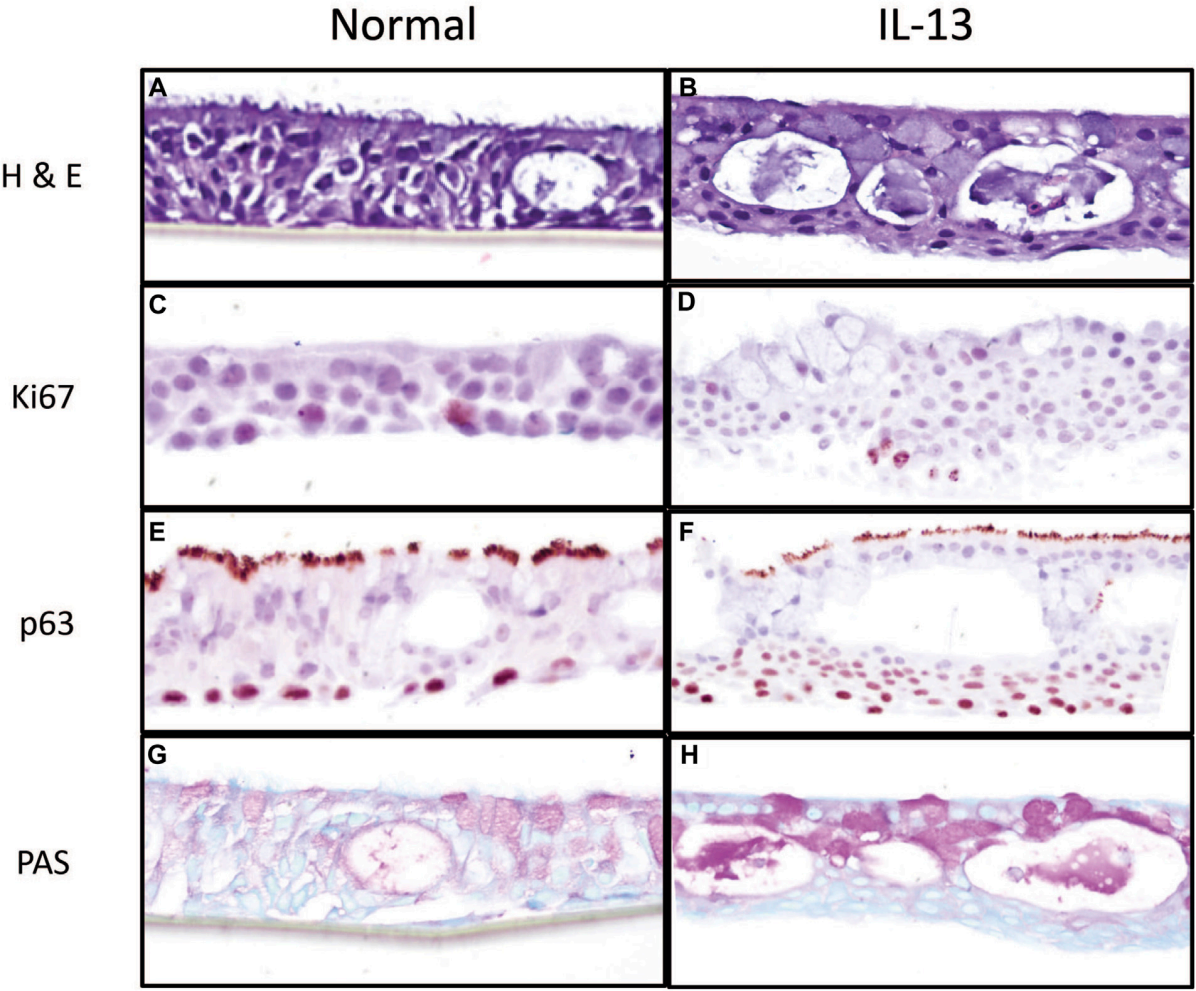
Morphological characterization of primary 3d human bronchial epithelial cells in culture with normal media and media containing IL-13 at 10 ng/mL. HBEC were fixed in 10% formalin and stained with H&E **(A, B)** to observe tissue structure, Ki67 **(C, D)** for actively proliferating cells, p63 **(E, F)** for basal cells, and PAS **(G, H)** to observe mucus and mucus-producing goblet cells. Images are shown at 20x magnification.

The morphology of differentiated HBEC cultures in the presence of normal media and media containing IL-13 was evaluated by histological methods prior to chemical exposure. H&E staining showed that cultures were fully differentiated into a pseudostratified mucocilliary epithelium showing cilia (Figures 1A,B). HBEC with the normal phenotype (Figure 1A) appeared to have more similar cell sizes and consistent tissue thickness compared to the IL-13 phenotype (Figure 1B), which showed varying cell sizes and tissue thickness. In addition, the IL-13 phenotype cultures appeared to show fewer (or shorter) cilia and more cytoplasmic vacuoles than normal cells. Immunohistochemistry was used to identify Ki67, a marker of actively proliferating cells, usually on the basal side (Figures 1C,D), and p63, a marker of basal epithelial cells (Figures 1E,F). The Ki67 staining was similar between the normal (Figure 1C) and IL-13 (Figure 1D) phenotypes; however, there were more layers of p63-positive basal cells in the IL-13 phenotype (Figure 1F) compared to the normal phenotype (Figure 1E). Mucus and mucus-producing goblet cells were visualized on the apical side with PAS staining (Figures 1G,H). There was an increased amount of mucus and mucus-producing cells in the IL-13 phenotype (Figure 1H) compared to normal (Figure 1G).

### 3.2 Barrier integrity and cytotoxicity in HBEC by IL-13 and BAP

**FIGURE 2:**
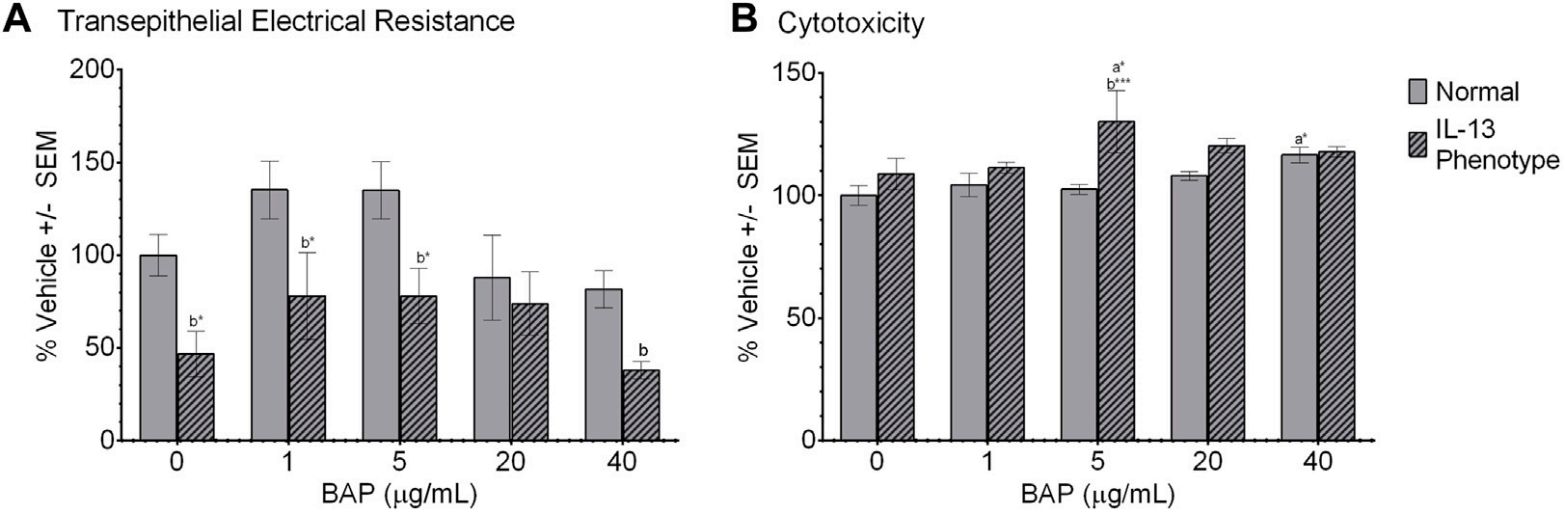
Barrier function and viability of HBEC after treatment. **(A)** Barrier integrity was measured as a difference in transepithelial electrical resistance (TEER) between apical and basal chambers. TEER values are calculated in ohms x cm^2^ (n = 4) and normalized to the DPBS treated group in the normal phenotype and scaled to 100%. **(B)** Cytotoxicity was measured as release of lactate dehydrogenase (LDH) from cells (n = 4) 48 h after treatment. LDH values were normalized to DPBS treated cells with the normal phenotype and scaled to 100%. **(A)** indicates significance compared to DPBS control of the normal phenotype. **(B)** indicates significance between the normal and IL-13 phenotypes within the same treatment group. Asterisks indicate level of significance (*p < 0.05, **p < 0.01, ***p < 0.001; one-way ANOVA with Fischer’s Least Significant Difference test). Comparison of TEER between normal and IL-13 phenotype at 40 μg/mL (*p* = 0.058).

We first investigated the effects of BAP on barrier integrity in normal and IL-13 phenotype cells, using TEER as a functional measure of barrier integrity. TEER was measured in ohms (Ω) x cm^2^ 48 h after BAP treatment and results were normalized to the DPBS treatment group in the normal phenotype (Figure 2A). The IL-13 phenotype resulted in significantly lower barrier integrity compared to the normal phenotype in both vehicle control and BAP treatment groups (Figure 2). In vehicle treated cells, TEER values in the normal phenotype were 158.15 ± 35.34 Ωcm^2^ compared to 73.92 ± 27.14 Ωcm^2^ in the IL-13 phenotype (average ±SE). This comparison between the phenotypes was significant (*p* < 0.05) at 0, 1 and 5 μg/mL BAP and approaching significance for 40 μg/mL BAP (*p* = 0.058). While there was no significant effect of BAP treatment compared to control cells, there was a trend towards decreased TEER values at the highest concentration (40 μg/mL BAP) tested in both phenotypes. Overall, these data show reduced barrier integrity in cells with the IL-13 phenotype.

Cytotoxicity was measured by the leakage of LDH in the cell media 48 h after exposure. All values were normalized to the DPBS control cells in the normal phenotype and scaled to 100% (Figure 2B). Overall, the effect of experimental conditions on cytotoxicity was minimal with no dose-dependent effect of BAP exceeding a 20% increase in cytotoxicity observed. There was a significant (*p* < 0.05) increase in LDH leakage in the normal phenotype when exposed to 40 μg/mL BAP compared to the DPBS control in the normal phenotype; although, this increase was only 20% greater than the control. In addition, there was a trend towards increased cytotoxicity in the IL-13 phenotype at concentrations of 5 μg/mL BAP and above, but a significant dose-dependent response was not observed.

### 3.3 Gene expression by quantitative PCR (qPCR)

**FIGURE 3:**
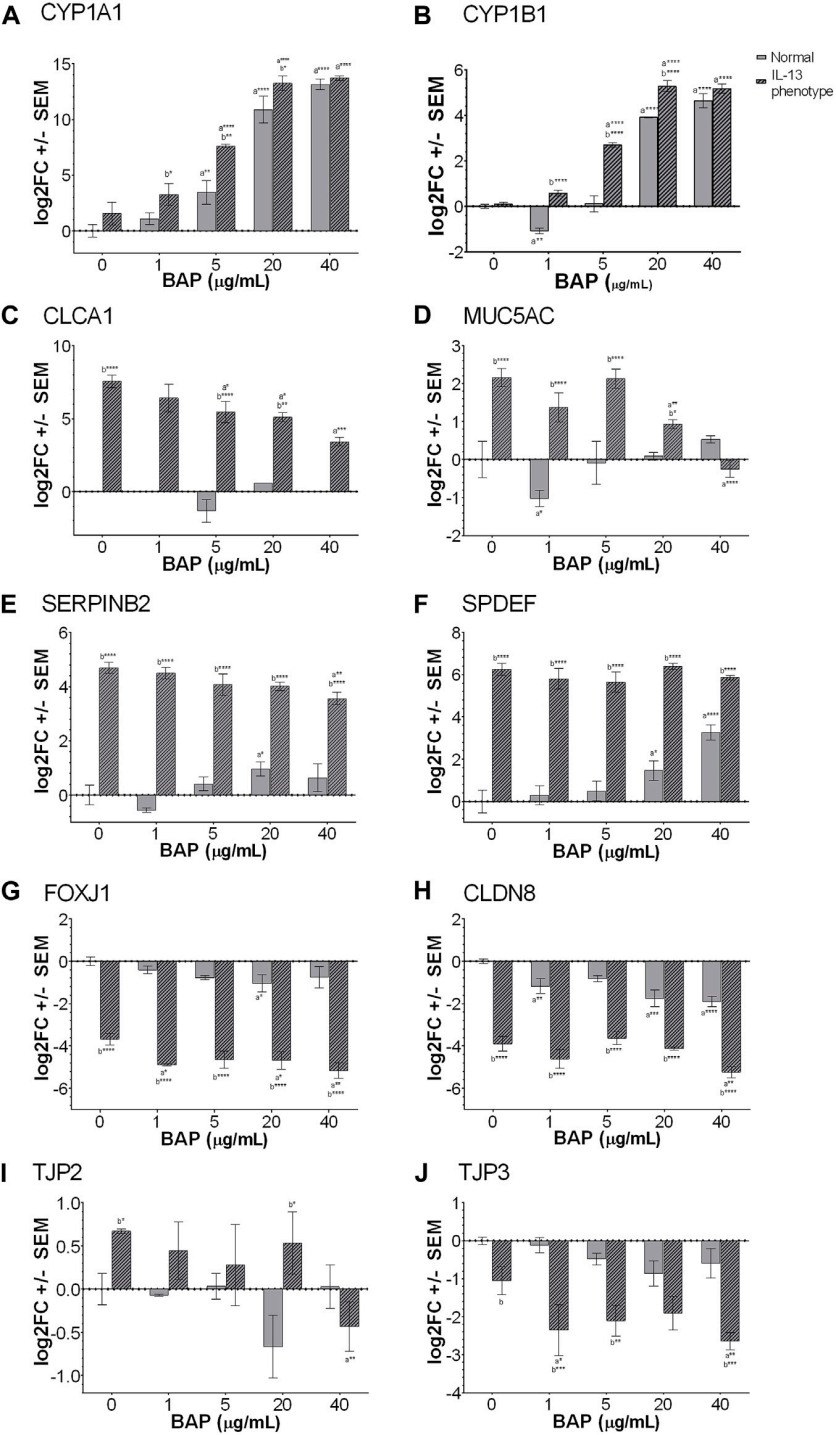
Expression of genes measured by qPCR (n = 4) for **(A)** *CYP1A1* **(B)** *CYP1B1* **(C)** *CLCA1* ^#^ **(D)** *MUC5AC* **(E)** *SERPINB2* **(F)** *SPDEF* **(G)** *FOXJ1* **(H)** *CLDN8* **(I)** *TJP2* and **(J)** *TJP3*. a indicates significance compared to DPBS of same phenotype. b indicates significance compared to BAP concentration is normal phenotype. Asterisks indicate level of significance (*p < 0.05, **p < 0.01, ***p < 0.001, ****p < 0.0001; one-way ANOVA with Fischer’s Least Significant Difference). There was a significant interaction between the effects of treatment and phenotype for CYP1B1, MUC5AC, SERPINB2 AND SPDEF (*p* < 0.01; two-way ANOVA). ^#^ *CLCA1* expression was undetectable in the majority of the normal phenotype groups, regardless of BAP exposure. Comparison of *TJP3* expression in the normal and IL-13 phenotype at 0 μg/mL (*p* = 0.0524).

Genes were selected for analysis by qPCR, including biomarkers associated with PAH exposure, the IL-13 induced asthmatic phenotype, and barrier integrity. The PAH biomarker genes selected for analysis by qPCR were Cytochrome P450 Family 1 Subfamily A Member 1 (*CYP1A1*) and Cytochrome P450 Family 1 Subfamily B Member 1 (*CYP1B1*) shown in Figures 3A,B. The *CYP1A1* and *CYP1B1* biomarkers resulted a significant (*p* < 0.05) dose-dependent increase with BAP exposure in both phenotypes. When comparing between the normal and IL-13 phenotype at the same BAP exposures, there was a significant increase in *CYP1A1* expression in the IL-13 phenotype at 1 μg/mL BAP (*p* < 0.05), 5 μg/mL BAP (*p* < 0.01), and 20 μg/mL BAP (*p* < 0.05). In addition, *CYP1B1* expression was similarly significantly (*p* < 0.05) increased in the IL-13 phenotype compared to normal cells at 1 μg/mL BAP (*p* < 0.0001), 5 μg/mL BAP (*p* < 0.0001), and 20 μg/mL BAP (*p* < 0.0001). There was a significant interaction between the effects of treatment and phenotype on CYP1B1 (*p* < 0.0001). To determine whether there is a shift in the dose-response curves for CYP450 induction between the normal and IL-13 phenotype, dose response curves were plotted with EC50 values (Supplementary Figure S1). Overall, the EC50 values for *CYP1A1* and *CYP1B1* were lower in the IL-13 phenotype (5.52 and 5.12 μg/mL) compared to the normal phenotype (11.02 and 12.57 μg/mL, respectively) suggesting that the asthmatic-induced phenotype may result in changes to AhR-mediated transcription that leads to greater potency for induction of biomarkers.

In order to investigate the development of the asthmatic phenotype, a panel of 4 different markers known to be associated with IL-13 and type 2 asthmatic inflammation were analyzed by qPCR (Figures 3C–F). Several markers, including chloride channel accessory 1 (*CLCA1*; Figure 3C), mucin 5AC (*MUC5AC*; 3D), serine protease inhibitor B2 (SERPINB2; Figure 3E), and SAM pointed domain containing ETS transcription factor (*SPDEF*; Figure 3F), are broadly related to type 2 inflammation, mucus production and goblet cell metaplasia (Mertens et al., 2017). These markers were all significantly (*p* < 0.001) increased in the IL-13 phenotype compared to normal cells confirming the IL-13 induced phenotype in untreated cells. In addition, there was a significant interaction between the effects of treatment and phenotype for MUC5AC (*p* < 0.0001), SERPINB2 (*p* < 0.01) AND SPDEF (*p* < 0.01). The interaction effect for CLCA1 was not able to be calculated due to lack of expression in the normal phenotype for most samples. However, after BAP exposure, *CLCA1*, *SERPINB2* and *MUC5AC* showed significantly (*p* < 0.05) decreased expression of transcripts with increasing concentration of BAP in the IL-13 phenotype compared to the DPBS IL-13 control cells indicating an effect of BAP treatment in the asthmatic model that was not observed in normal cells. In normal cells, BAP treatment resulted in no significant changes or minimal changes to *CLCA1*, *SERPINB2* and *MUC5AC* transcript levels that were not dose-dependent. For the marker *SPDEF*, BAP treatment in normal cells resulted in a dose-dependent increase (*p* < 0.05) in transcript expression. No effect of BAP was observed for *SPDEF* in the IL-13 phenotype; although, this marker may already have been expressed to a maximal extent in untreated cells making it difficult to observe an effect.

Another biomarker, forkhead box J1 (*FOXJ1*; Figure 3G) regulates the transcription of genes related to cilia gene expression (Gomperts et al., 2007). Our data show that in the normal phenotype, *FOXJ1* expression is slightly downregulated when exposed to BAP, only showing a significant decrease in expression when exposed to 20 μg/mL BAP (*p* < 0.05). In the IL-13 phenotype, there was a significant (*p* < 0.0001) decrease in *FOXJ1* expression in untreated cells compared to the normal phenotype. When the IL-13 phenotype samples were exposed to BAP, expression of *FOXJ1* was further decreased at 1 μg/mL (*p* < 0.05), 20 μg/mL (*p* < 0.05), and 40 μg/mL (*p* < 0.01), relative to the IL-13 phenotype control treatment group indicating a dose-dependent effect between BAP treatment in the asthmatic model that was not observed in normal cells.

In order to investigate barrier integrity, the transcriptional markers claudin 8 (*CLDN8*; Figure 3H), tight junction protein 2 (*TJP2*; Figure 3I), and tight junction protein 3 (*TJP3*, Figure 3J) were analyzed by qPCR (Rezaee and Georas, 2014; Chang et al., 2019; Schmidt et al., 2019). *CLDN8* and *TJP3*, which are important for establishment of tight junctions, were both decreased in the IL-13 phenotype compared to normal HBEC in untreated cells (*p* < 0.0001 for *CLDN8*; *p* = 0.0524 for *TJP3*). After BAP treatment in the IL-13 phenotype, both *CLDN8* and *TJP3* expression were further decreased (*p* < 0.05) with increasing concentrations of BAP compared to the IL-13 control indicating an effect with BAP treatment in the asthmatic model. In the normal phenotype, BAP also caused some decrease in *CLDN8* and *TJP3* expression that was only significant for CLDN8 (*p* < 0.01). *TJP2* is a gene that plays an important role in the maintenance and retention of tight junctions. There was no significant change in *TJP2* expression in the normal phenotype after treatment with BAP (Figure 3I). In the samples with the IL-13 phenotype, expression of *TJP2* was significantly (*p* < 0.05) increased in the untreated control compared to normal cells and significantly decreased after BAP treatment at the highest concentration of 40 μg/mL (*p* < 0.01) suggesting a similar effect of BAP in the asthmatic model as observed for *CLDN8* and *TJP3*.

### 3.4 Global expression

**FIGURE 4:**
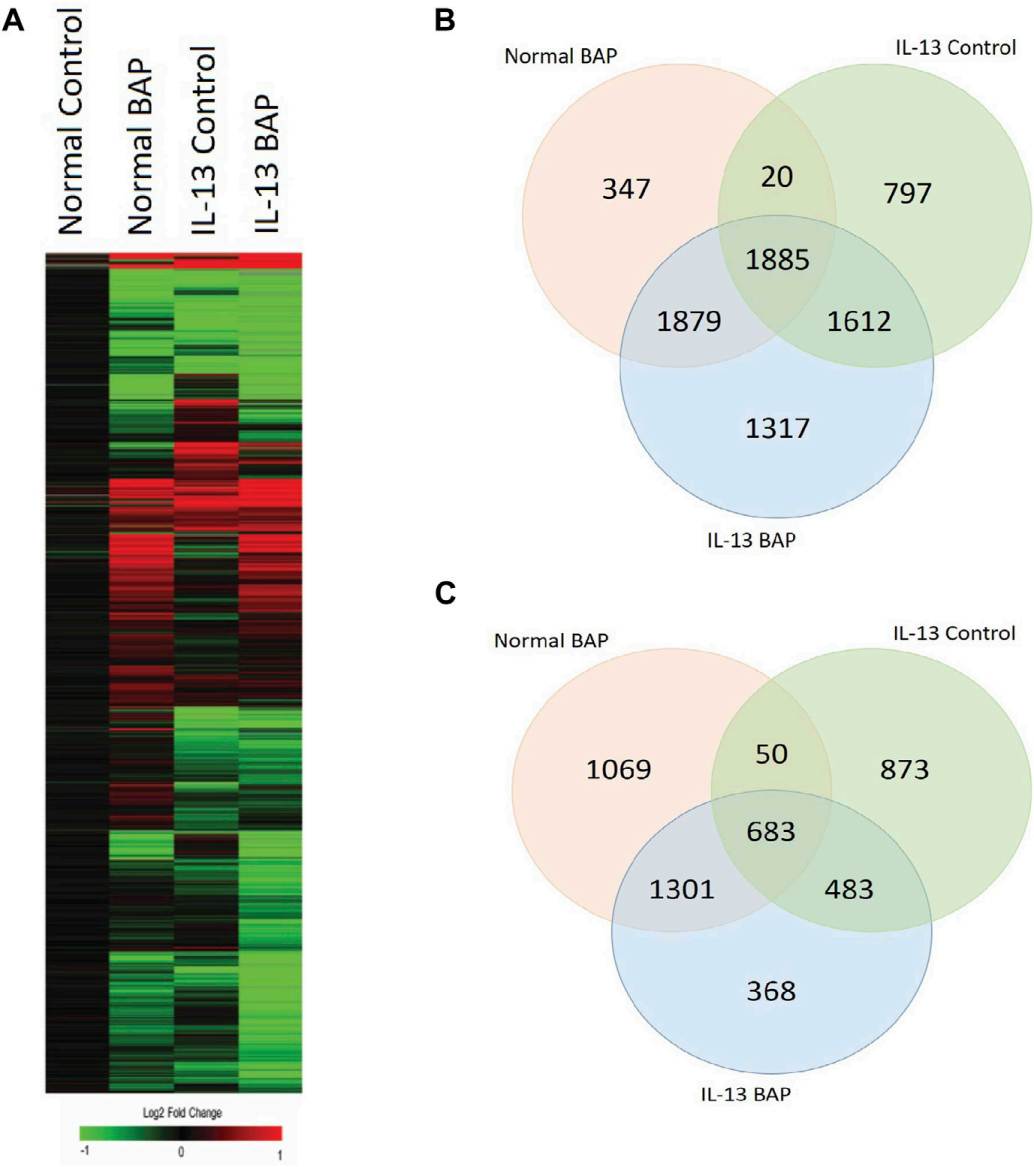
HBEC transcriptional response to BAP in normal HBEC compared to IL-13 phenotype. Global gene expression was measured after 48 h exposure to 40 μg/mL BAP or DPBS vehicle in HBEC (n = 4) with normal or IL-13 phenotype. **(A)** Hierarchical clustering of genes differentially expressed (|log2 fold change| > 0.58; q < 1 × 10^−7^) by BAP, IL-13, and BAP-IL13 compared to vehicle control. Red, green, and black represent positive, negative, and unchanged genes, respectively. **(B)** Venn diagram of significantly increased transcripts (log2 fold change >0.58; q < 1 × 10^−7^) in each treatment group compared to the normal vehicle control group. **(C)** Venn diagram of significantly decreased transcripts (log2 fold change < −0.58; q < 1 × 10^−7^) in each treatment group compared to the normal vehicle control group.

We investigated the effect of the highest concentration BAP treatment (40 μg/mL) on 3D HBEC with the normal and IL-13 phenotype by analyzing global gene expression patterns in RNA sequencing data. Raw and normalized sequencing files are available online at NCBI Gene Expression Omnibus (GSE239797). There were 12,275 statistically significant (q < 1 × 10^−7^, |log2FC|>0.58) expressed genes across all treatments compared to normal control cells. A heatmap of the differentially expressed genes (DEG) was generated using a hierarchical clustering analysis (Figure 4A). Transcripts that were increased and decreased compared to control were organized into a venn diagram to compare gene regulation across all conditions (Figures 4B,C). BAP treatment in cells with the normal phenotype resulted in 3,103 significantly increased transcripts (1,069 unique to BAP treatment) and 4,131 significantly decreased transcripts (347 unique to BAP treatment). Vehicle control cells with the IL-13-induced asthmatic phenotype, referred to as IL13, resulted in 2,089 significantly increased transcripts (873 unique to IL13) and 4,314 decreased transcripts (797 unique to IL13). The cells with the IL-13-induced asthmatic phenotype and treated with BAP, referred to as BAP-IL13, resulted in 2,835 increased transcripts (368 unique to BAP-IL13) and 6,693 significantly decreased transcripts (1,317 unique to BAP-IL13). Approximately half, or 54%, of all differentially expressed genes in the study were decreased in the BAP-IL13 group, 11% of which are uniquely decreased in the BAP-IL13 group only. Gene expression analysis supports the hypothesis that the IL-13 induced asthmatic phenotype responds uniquely to chemical insult by BAP exposure compared to normal HBEC.

**FIGURE 5:**
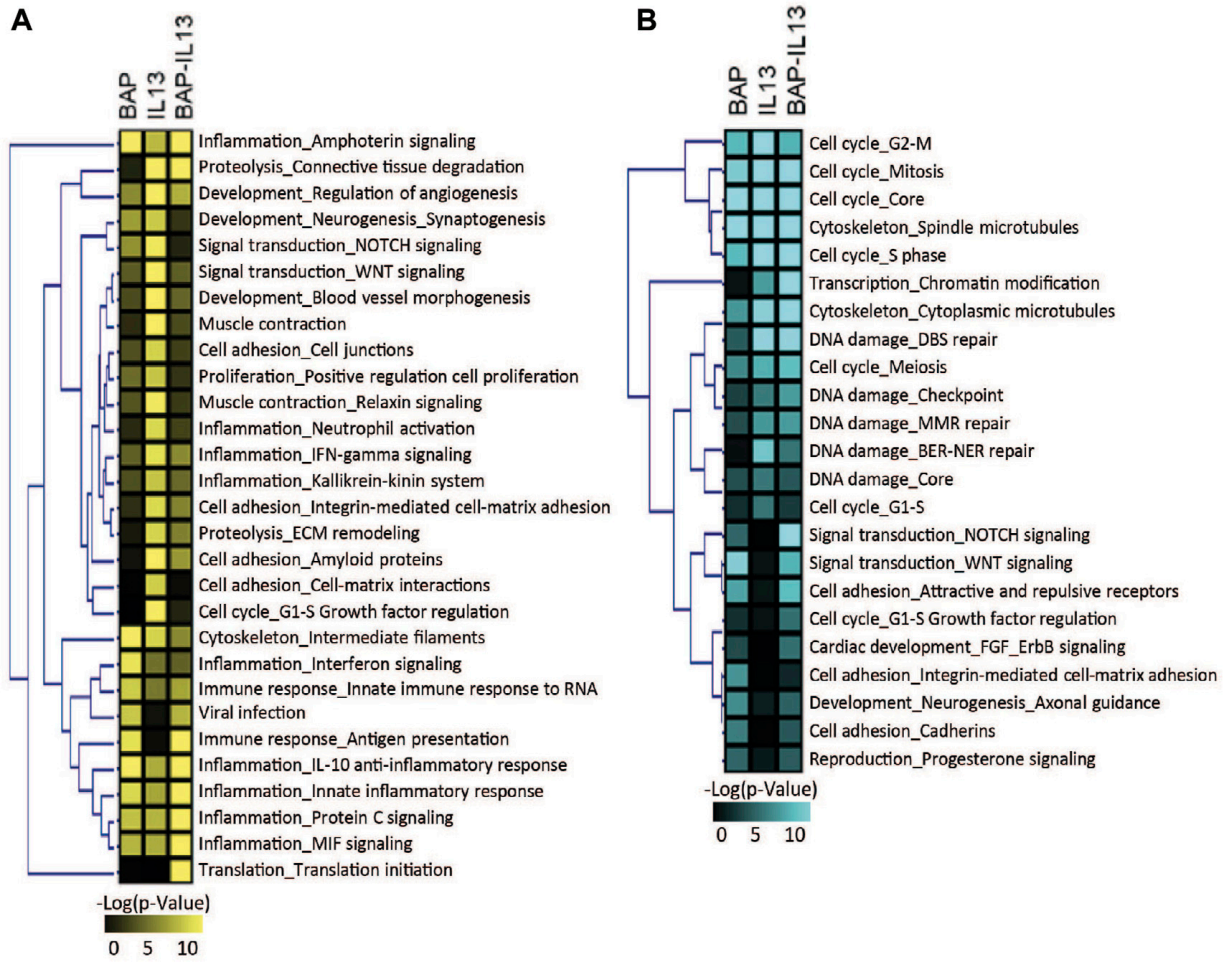
Biological processes significantly (*p* < 1 × 10^−5^) enriched in HBEC after treatment with 40 μg/mL BAP, after development of the IL-13 phenotype and after treatment of IL-13 HBEC with BAP compared to normal HBEC treated with DPBS vehicle control. Heatmap shows pathways significant for **(A)** increased transcripts and **(B)** decreased transcripts. Significance increases with color in both heatmaps.

To further understand which biological processes were being regulated across all conditions, transcripts that were increased and decreased in the BAP, IL13, and BAP-IL13 groups compared to the normal phenotype control were analyzed separately for enriched pathways. These data were analyzed to identify the most significant pathways regulated in each treatment group. All processes that meet the criteria of p < 1E-5are included in Figure 5. For the IL-13 phenotype, some of the most significantly increased pathways included cell adhesion and cell-matrix interactions, proteolysis and connective tissue degradation, ECM remodeling, WNT signaling and cell proliferation (Figure 5A). The most significantly decreased pathways included cell cycle processes (*p* = 3.1E-16), DNA damage (1.46E-5), cytoskeleton (8.32E-16) and chromatin modification (3.57E-7) (Figure 5B). For the BAP-treated normal HBEC, increased processes included several inflammatory signaling processes, cytoskeleton and apoptosis (*p* = 1.32E-4), while decreased processes included those related to cell cycle, cell adhesion and WNT/NOTCH signaling. For the IL-13 phenotype treated with BAP, a large number of inflammatory processes were significantly increased, while processes for cell cycle and DNA damage were decreased.

**FIGURE 6:**
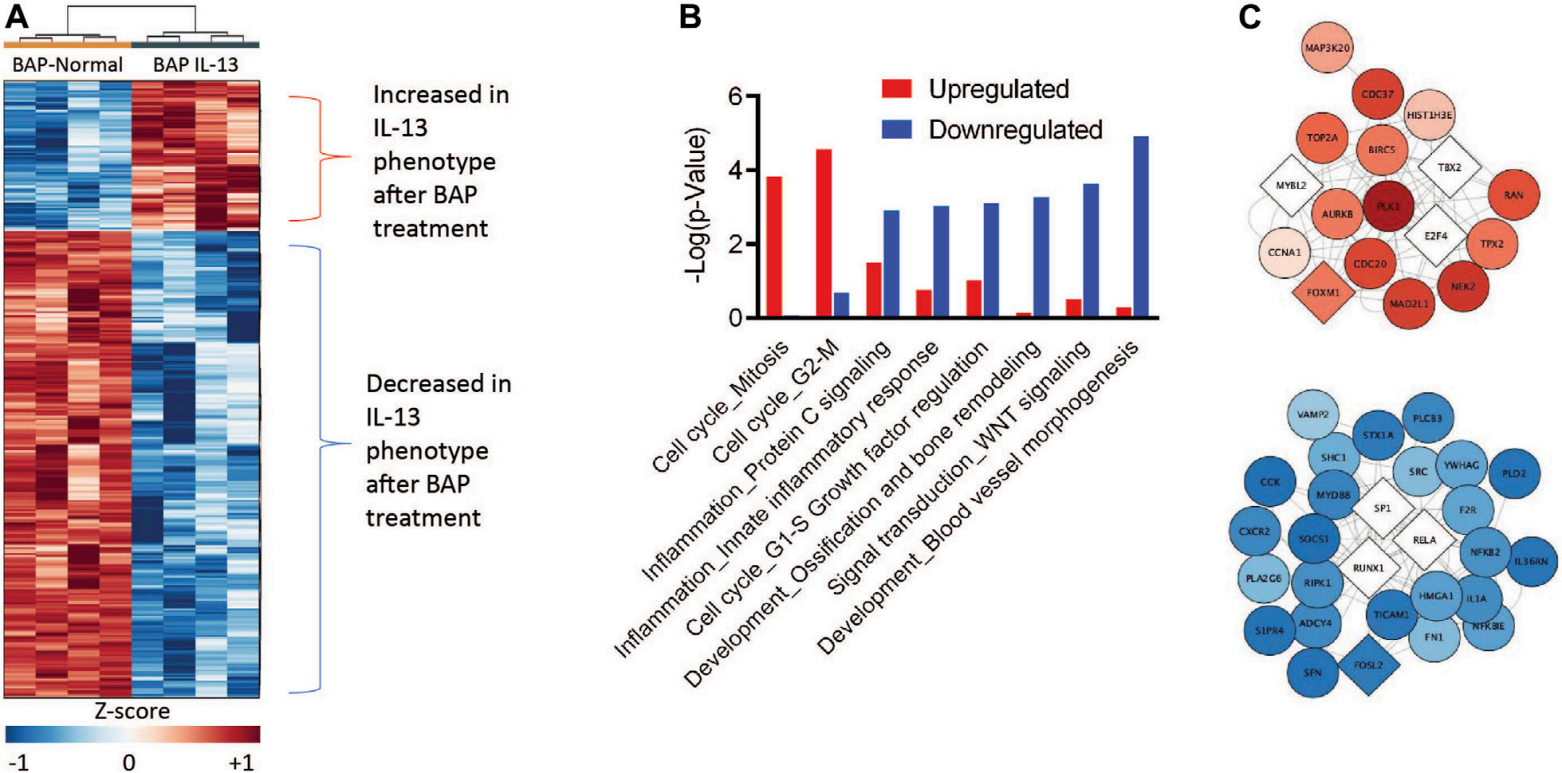
Comparison of gene expression in HBEC with the IL-13 phenotype compared to normal phenotype after treatment with 40 μg/mL BAP (n = 4) normalized to their respective phenotype controls. **(A)** Hierarchical clustering of 971 significantly expressed genes. Values in heatmap are z-score transformations across BAP/Control and BAP-IL13/IL13 groups. Red, blue, and white represent positive, negative, and unchanged genes, respectively. Insets show the networks for genes up- or downregulated by asthmatic phenotype. Darker red or blue genes indicate more increased or decreased expression relatively to the normal phenotype, respectively. **(B)** Functional enrichment of processes by BAP exposure in normal and asthmatic phenotype HBEC using MetaCore (GeneGo). Red bars indicate increase in the asthmatic phenotype relative to normal samples. Blue bars indicate decrease in the asthmatic phenotype relative to normal samples. **(C)** Network interaction figures for cell cycle and inflammation processes. Circular nodes represent significant genes and diamond nodes represent enriched transcription factors. The color of the nodes indicates the average z-score of the IL-13 phenotype samples after BAP exposure compared to the normal phenotype samples after BAP exposures. The intensity of the red and blue nodes indicate more extreme positive and negative z-score values, respectively, on average in the IL-13 phenotype compared to the normal phenotype after BAP exposure.

A primary goal of this study is to understand how the IL-13 phenotype responds to chemical insult compared to normal HBEC. Therefore, to identify the transcripts and biological processes that are the most different in the IL-13 phenotype compared to the normal phenotype after BAP treatment, we focused on the 971 genes differentially expressed (q < 1 × 10^−7^, |log2FC|>0.58) between the BAP-treated normal phenotype and the BAP-treated IL-13 phenotype. A z-score analysis was used to visualize these genes in a heatmap after hierarchical clustering (Figure 6A). Of those genes, 236 were significantly increased after BAP exposure in the presence of the IL13 induced asthmatic phenotype relative to the normal phenotype and 735 genes were significantly decreased after BAP exposure in the IL13 induced asthmatic phenotype relative to the normal phenotype. Differentially expressed transcripts that were increased and decreased in the IL-13 phenotype compared to normal HBEC after BAP treatment were analyzed for significantly (*p* < 0.01) enriched pathways (Figure 6B). Two cell cycle processes were significantly upregulated in the IL13 induced asthmatic phenotype relative to the normal phenotype after BAP exposure while 22 pathways associated with various processes such as inflammation, development, and signal transduction were significantly downregulated in the IL13 induced asthmatic phenotype. All significantly regulated genes from upregulated cell cycle processes and downregulated inflammation processes and present in the z-score analysis data were compiled into two respective lists. Each compiled list was used to identify the three most significant (*p* < 3.8E-10 for cell cycle; *p* < 7.3E-7 for inflammation) transcription factors associated with those genes in the MetaCore database. Interactions between genes and transcription factors were determined in MetaCore. The interactions and calculated z-score for genes were used to create cell cycle and inflammation networks (Figure 6C). Overall, these data suggest that HBEC with the IL-13 induced asthmatic phenotype have increased potential for uncontrolled proliferation and decreased inflammatory response after treatment with BAP.

## 4 Discussion

This study utilized an *in vitro* differentiated 3D HBEC model to investigate how individuals with type 2 inflammation associated with asthma respond to chemical insult after PAH exposure. In addition, this study explores the mechanisms associated with PAH toxicity in an IL-13 induced asthmatic phenotype compared to normal HBEC. The asthmatic phenotype, in which HBEC were differentiated in the presence of IL-13, was characterized by histology and evaluated for biomarkers of cytotoxicity, barrier integrity, mucus production, cell differentiation, cilia development, type 2 inflammation and PAH metabolism in the presence and absence of BAP treatment compared to normal HBEC. Global gene expression by RNA sequencing was analyzed in order to identify differences in mechanistic response to BAP driven by the normal and IL-13 phenotype in HBEC.

### 4.1 Characterization of the asthmatic phenotype

IL-13 is a primary driver in the type 2 inflammation associated with allergic asthma (Maspero et al., 2022). Type 2 inflammation in allergic asthma is associated with symptoms such as impaired lung function, wheezing, and shortness of breath, among other symptoms. The role of IL-13 in type 2 inflammation is associated with goblet cell hyperplasia, mucociliary dysfunction, excess mucus production, airway remodeling, and epithelial barrier dysfunction. Exposure to IL-13 has been shown to decrease barrier integrity as measured by TEER and transcriptional markers in primary and immortalized HBEC (Parker et al., 2013; Saatian et al., 2013; Georas and Rezaee, 2014). Additionally, IL-13 has been shown to cause mucus hypersecretion, contributing the pathophysiology of allergic asthma, as measured by MUC5AC gene expression (Zhen et al., 2007).

In the present study, TEER measurements were taken as a functional measure of barrier integrity. Our data show a significant decrease in TEER (*p* < 0.05) in IL-13 phenotype samples compared to normal samples after exposure to vehicle control. Additionally, we evaluated several markers of barrier function including TJP3 and CLDN8 by qPCR. TJP3 and CLDN8, which contribute to formation of tight junctions and their permeability, had decreased expression which supported the development of the asthma-like phenotype. Alternatively, TJP2, a marker associated with maintenance of tight junctions was significantly (*p* < 0.05) increased in the IL-13 phenotype, suggesting that HBEC with an asthma-like phenotype may only be able to maintain previously developed tight junctions.

Our histological images showed an increase in basal cell layers of epithelium which is consistent with findings in tissues from individuals with severe asthma (Cohen et al., 2007). The thickening of the airway has been suggested to result in or contribute to airway hyperresponsiveness. PAS staining also indicated an increase in mucus and mucus-producing goblet cells, which is consistent with the reported effects of IL-13 and type 2 inflammation in asthma and other airway diseases (Maspero et al., 2022).

Additionally, we further evaluated development of the asthmatic phenotype by analyzing mucus production since mucus production and secretion has been a hallmark of asthma and has been known to contribute to the airway hyperresponsiveness associated with the disease (Evans et al., 2009). Our data showed significant (*p* < 0.0001) increase in MUC5AC expression in asthmatic phenotype samples compared to normal samples after vehicle control exposure. Mucociliary dysfunction was further confirmed by significant (*p* < 0.0001) dysregulation of FOXJ1, a gene known to contribute to cilia formation and development (Tilley et al., 2015). Other markers previously associated with type 2 inflammation in asthma, including CLCA1, SERPINB2, and SPDEF, were significantly (*p* < 0.0001) increased in the IL-13 treated HBEC compared to normal HBEC (Mertens et al., 2017; Pavlidis et al., 2019; Jackson et al., 2020).

TEER measurements and MUC5AC expression evaluated through qPCR support the development of the asthmatic phenotype after cellular differentiation in the presence of IL-13 for 2 weeks.

### 4.2 BAP interactions with the normal and asthmatic phenotypes

An important goal of this study is to determine whether HBEC expressing the IL-13 phenotype respond differently to PAH exposure than normal HBEC indicating that asthmatic individuals may be more susceptible to toxicity. It has previously shown that BAP and other PAHs can disrupt barrier integrity in *vitro* bronchial epithelium, in addition to effecting biological processes of cell cycle, oxidative stress and DNA damage (Chang et al., 2019; 2020). The disruption in barrier integrity can cause dysregulation of inflammation and oxidative stress (Schamberger et al., 2014), ultimately leading to increased airway inflammation suggesting the potential for an combined effect after PAH exposure in individuals with pre-existing lung inflammation (Coyne et al., 2002). While functional measures of barrier integrity resulted in no significant changes in the normal phenotype after BAP exposure in this study, TEER values were consistently decreased in the IL-13 phenotype compared to normal (Figure 2) supporting the relationship between the inflammation and barrier integrity in the lung (Devalia et al., 1997). The effect of BAP in the IL-13 phenotype was more apparent when evaluating transcriptional biomarkers of barrier integrity in which all markers tested, CLDN8, TJP2 and TJP3, were significantly (*p* < 0.05) decreased in the IL-13 phenotype after treatment with BAP compared to the IL-13 control (Figure 4) indicating an increased sensitivity to reduced barrier integrity BAP in the asthmatic phenotype. Reduced barrier integrity may make individuals more susceptible to adverse health outcomes from environmental factors such as particulate matter, pathogens, and contaminants. This is due to the reduced capability of maintaining airway homeostasis and weakened physical barrier that the bronchial epithelium creates (Sözener et al., 2020).

In HBEC, normal mucus production is important for maintaining the epithelial barrier. Normal airway mucus secretion strengthens the airway barrier by first interacting with inhaled pollutants (Sözener et al., 2020). Particles and pollutants inhaled may be trapped in mucus which is then cleared by cilia, creating a normal mucociliary escalator (Knowles and Boucher, 2002; Davies, 2014). Previous studies had shown that MUC5AC expression was induced after BAP exposure in *vitro* lung epithelial cells and *in vivo* murine studies (Y. Sun et al., 2021; Sun et al., 2022). For several markers related to mucus production, goblet cell hyperplasia and type 2 inflammation (Figure 4), we observed a dose-dependent decrease in the IL-13 phenotype after BAP treatment indicating that BAP causes a reduction in mucus production and inflammation in the presence of the asthmatic phenotype that is not observed in normal HBEC. In addition, there was a significant interaction effect between chemical treatment and IL-13 induced asthmatic phenotype for these endpoints. The excessive mucus produced during airway disease can lead to congestion and restricted breathing, but normal amounts of mucus production are important for trapping inhaled toxicants and deficient mucous barrier can leave the lungs vulnerable to injury from chemical, particulate, and pathogen insults (Fahy and Dickey, 2010).

It is well studied that PAHs are ligands of the aryl hydrocarbon receptor, which is a key regulator of cytochrome P450 expression, including CYP1A1 and CYP1B1 (Trombino et al., 2000; Ye et al., 2019). Induction of CYP1A1 and CYP1B1 is specifically associated with metabolizing BAP to ultimately produce a diol-epoxide which can lead to the development of cancer (Klingbeil et al., 2014). We have also previously seen that exposure to PAHs, including BAP, induces expression of *CYP1A1* and *CYP1B1* in normal HBEC (Chang et al., 2019; Chang et al., 2020). The *CYP1A1* and *CYP1B1* expression data suggests an increased sensitivity of the IL-13 phenotype to chemical treatment, which was significant for CYP1B1. Prior studies have reported on potential of complex interactions between inflammation-based disease and cytochrome P450 enzyme activity suggesting an interaction between signaling pathways (de Jong et al., 2020). Overall, our data suggests that BAP interacts with the asthmatic phenotype in significantly different ways than the normal phenotype that may alter sensitivity to BAP exposures.

### 4.3 Altered BAP response in asthmatic phenotype

To understand which mechanisms are contributing to the unique response in the IL-13 phenotype compared to the normal phenotype after exposure to BAP, we evaluated the biological processes for genes that are the most differentially expressed between the two phenotypes after treatment. A relatively small portion of genes significantly regulated after z-score analysis were significantly upregulated in the asthmatic phenotype after BAP exposure relative to the normal phenotype. These genes were only significantly involved in processes related to cell cycle regulation. Furthermore, these genes were associated with enriched transcription factors including T-Box Protein 2 (*TBX2*), E2 Transcription Factor 4 (*E2F4*), and MYB Proto-Oncogene 2 (*MYBL2*). Enriched transcription factor Forkhead Box M1 (*FOXM1*) was also present. Dysregulation of these 4 transcription factors is associated with uncontrolled proliferation in various phases of the cell cycle. Prior *in vivo* studies have shown that TBX2 regulated expression is critical for normal development of epithelial cells from lung mesenchyme (Lüdtke et al., 2013; Lüdtke et al., 2021). Particularly, TBX2 is known to repress p21, or CDKN1A, which is a negative cell cycle regulator (Lüdtke et al., 2013). Inhibited expression of this negative cell cycle regulator may result in uncontrolled proliferation. Similarly, the E2F family is a family of transcription factors generally associated with cell cycle regulation. The E2F family is a part of the highly conserved DREAM complex that plays a crucial role in cell cycle regulation, particularly during the G0 phase (Sadasivam and DeCaprio, 2013). Dysregulation of the DREAM complex can result in a shift from quiescence to proliferation. E2F4 is canonically a tumor suppressor gene; however, loss of E2F4 in progenitor cells of various tissues has been associated with decreased proliferation (Hsu and Sage, 2016). In HBECs specifically, little research has been done to clarify the specific role of E2F4. A previous study showed that E2F4 expression is associated with ciliated cell differentiation and normal airway epithelium development in a murine model but had little impact on cell cycle progression and dysregulation (Danielian et al., 2007).

The third transcription factor identified in the upregulated network, MYBL2, is a cell cycle regulator and in part regulated by the DREAM complex (Sadasivam and DeCaprio, 2013). When MuvB, a component of the DREAM complex, binds to MYBL2, there may be disassembly of the DREAM complex. Additionally, it is proposed that MYBL2 may be a part of a positive feedback loop. As expression of MYBL2 increases, it may promote further disruption of DREAM and its transcriptional regulation (Iness et al., 2019). Overall, this suggests that overexpression of MYBL2 has the potential to result in uncontrolled proliferation. Lastly, *FOXM1* expression has been associated with asthma pathogenesis. It has particularly been associated with induction of Type 2 lung inflammation and goblet cell metaplasia (Li et al., 2019). In addition to its role in asthma pathogenesis, FOXM1 has been identified as a subsequent target for the MuvB-MYBL2 complex with important roles in mitosis (Sadasivam et al., 2012; Sadasivam and DeCaprio, 2013). Overall, the presence of these 4 enriched transcription factors in a network of genes relatively upregulated in the asthmatic phenotype after BAP exposure compared to the normal phenotype suggests that there may be increased susceptibility to BAP resulting in uncontrolled proliferation in the asthmatic phenotype. Uncontrolled proliferation in the asthmatic phenotype following BAP exposure may lead to decreased lung function as the airway wall thickens and results in potential increase in airway obstruction and hyperresponsiveness (Cohen et al., 2007).

A large proportion of transcripts were significantly decreased in the IL13 phenotype after BAP treatment relative to the normal phenotype, many of which were associated with processes of inflammation and regulation through the transcription factor NF-kB (Figure 6B). These genes and transcription factors include Runt-related Transcription Factor 1 (*RUNX1*), NF-kB subunit p65 (*RELA*), and NF-kB subunit 2 (*NFKB2*). The functions the NF-kB pathway have been well studied. Normal expression and activation of NF-kB subunits is essential for normal development and regulation of innate and adaptive immunity, as well as cell survival (Aggarwal et al., 2004). Dysregulation of NF-kB signaling, which can lead to chronic inflammation and disruption of homeostasis, has been associated with a variety of diseases including asthma (Yu et al., 2020). Many of the proinflammatory molecules, such as cytokines and adhesion molecules, associated with asthma are regulated by NF-kB (Christman et al., 2000; Yamamoto and Gaynor, 2001). Furthermore, activation of NF-kB has been identified in critical areas of asthmatic lungs (Christman et al., 2000). The function of RUNX1 in respiratory epithelium cells have not been fully established and few studies fully investigate RUNX1’s role. However, the limited data available shows that loss of RUNX1 expression increases NF-kB activation through reduced attenuation of NF-kB inhibitors (Tang et al., 2017; Tang et al., 2017).

NF-kB signaling is directly impacted by the regulation of RELA and NFKB2, as these are two subunits of NF-kB. RELA is a well-established subunit involved in the canonical pathway of NF-kB signaling and activation and plays a role in all immune responses (S.-C. Sun, 2017; Yu et al., 2020). RELA has previously been shown to associate with AhR resulting in the repression of NF-kB pathways (Tian et al., 1999). Other studies have also shown that dysregulation of AhR and NF-kB subunits result in changes in cytokine expression (Ishihara et al., 2019). Alternatively, NFKB2, which is also known as p52 or p105, has been implicated as a non-canonical pathway subunit and has a more specific role in the immune system and immune responses (S.-C. Sun, 2017; Yu et al., 2020). The relative decrease in expression of these two subunits impacts both the canonical and non-canonical pathways of NF-kB and ultimately disrupts homeostasis and immune responses to stimuli such as BAP. While genes and transcription factors associated with the inflammation pathway may have been upregulated in the asthmatic phenotype compared to the normal phenotype, they were relatively decreased in the asthmatic phenotype after BAP exposure compared to the normal phenotype after BAP exposure. This ultimately suggests that there is a decreased ability for cells with the asthmatic phenotype to respond to chemical insult.

## 5 Conclusion

In summary, this work demonstrated the utilization of an asthmatic phenotype in primary human bronchial epithelial cells cultured at the ALI to further investigate how individuals with pre-existing conditions may respond differently to chemical stimuli, such as BAP. We were able to confirm the development of the phenotype through reduced barrier integrity, increased expression of asthmatic markers, and increased presence of goblet cells. After exposure to BAP, our data shows a decrease in the ability of HBEC to respond to chemical insult through decreased metabolic capacity and a relative decrease in immune responses like inflammation. Additionally, our data showed a potential for more uncontrolled proliferation in the asthmatic phenotype after BAP exposure.

## Data Availability

The datasets presented in this study can be found in online repositories. The names of the repository/repositories and accession number(s) can be found below: https://www.ncbi.nlm.nih.gov/geo/, GSE239797.
