# Supplementary material for: Assessing susceptibility for polycyclic aromatic hydrocarbon toxicity in an in vitro 3D respiratory model for asthma
Source: Front Toxicol. 2024 Apr 19;6:1287863. doi: 10.3389/ftox.2024.1287863 (PMC11066177; doi:10.3389/ftox.2024.1287863)

Supplemental Data File

Assessing susceptibility for polycyclic aromatic hydrocarbon toxicity in an *in vitro* 3D respiratory model for asthma

Reese M. Valdez[a,b], Brianna N. Rivera[a,b], Yvonne Chang[a,b], Jamie M. Pennington[a], Kay A. Fischer[c], Christiane V. Löhr[c], Susan C. Tilton[a,b]

[a] *Environmental and Molecular Toxicology Department, Oregon State University, Corvallis, OR, USA*

[b] *Superfund Research Program, Oregon State University, Corvallis, OR, USA*

[c] *College of Veterinary Medicine, Oregon State University, Corvallis, OR, USA*

Supplemental Table 1. Primer sequences with gene name and NCBI gene ID used in present study.

Gene	Primer Sequence	NCBI Gene ID	Reference
PPIA	F: GCATACGGGTCCTGGCATCTTGTCC	5478	Chang et al, 2019
	R: ATGGTGATCTTCTTGCTGGTCTTGC		
CYP1A1	F: TCGGCCACGGAGTTTCTTC	1543	Chang et al, 2019
	R: GGTCAGCATGTGCCCAATCA		
CYP1B1	F: CCAACCTGCCCTATGTCCT	1545	Chang et al, 2019
	R: CTGGATCAAAGTTCTCCGGG		
CLCA1	F: ATGGCTATGAAGGCATTGTCG	1179	Mertens et al, 2017
	R: TGGCACATTGGGGTCGATTG		
FOXJ1	F: ACTCGTATGCCACGCTCATCTG	2302	Salgueiro et al, 2022
	R: GAGACAGGTTGTGGCGGATTGA		
MUC5AC	F: CCTTCGACGGACAGAGCTAC	4586	Mertens et al, 2017
	R: TCTCGGTGACAACACGAAAG		
SERPINB2	F: TCCTGGGTCAAGACTCAAACC	5055	Mertens et al, 2017
	R: CATCCTGGTATCCCCATCTACAG		
SPDEF	F: ATGAAAGAGCGGACTTCACCT	25803	Mertens et al, 2017
	R: CTGGTCGAGGCACAGTAGTG		
CLDN8	F: CAACCCATGCCTTAGAAATCGC	9073	Koumangoye et al, 2022
	R: TCACGCAATTCATCCACAGTC		
TJP2	F: GGCCTACGACCCAGACTAC	9414	Chang et al, 2019
	R: ACTCTTCGTTCGCTCTGCTTT		
TJP3	F: GCTTCCTCAAGGGCAAGAGCAT	27134	Park et al, 2023
	R: CGTGTCAGGTTCTGGAATGGCA		

Chang, Y., Siddens, L. K., Heine, L. K., Sampson, D. A., Yu, Z., Fischer, K. A., Löhr, C. V., & Tilton, S. C. (2019). Comparative mechanisms of PAH toxicity by benzo[a]pyrene and dibenzo[def,p]chrysene in primary human bronchial epithelial cells cultured at air-liquid interface. *Toxicology and Applied Pharmacology*, *379*, 114644. https://doi.org/10.1016/j.taap.2019.114644

Koumangoye, R., Penny, P., & Delpire, E. (2022). Loss of NKCC1 function increases epithelial tight junction permeability by upregulating claudin-2 expression. *American Journal of Physiology - Cell Physiology*, *323*(4), C1251–C1263. https://doi.org/10.1152/ajpcell.00334.2022

Mertens, T. C. J., van der Does, A. M., Kistemaker, L. E., Ninaber, D. K., Taube, C., & Hiemstra, P. S. (2017). Cigarette smoke differentially affects IL-13-induced gene expression in human airway epithelial cells. *Physiological Reports*, *5*(13), e13347. https://doi.org/10.14814/phy2.13347

Park, S.-H., Lee, H.-C., Jeong, H. M., Lee, J.-S., Cha, H.-J., Kim, C. H., Kim, J., & Song, K. S. (2023). Inhibition of Urban Particulate Matter-Induced Airway Inflammation by RIPK3 through the Regulation of Tight Junction Protein Production. *International Journal of Molecular Sciences*, *24*(17), Article 17. https://doi.org/10.3390/ijms241713320

Salgueiro, L., Kummer, S., Sonntag-Buck, V., Weiß, A., Schneider, M. A., Kräusslich, H.-G., & Sotillo, R. (2022). Generation of Human Lung Organoid Cultures from Healthy and Tumor Tissue to Study Infectious Diseases. *Journal of Virology*, *96*(7), e00098-22. https://doi.org/10.1128/jvi.00098-22

Supplemental Figure 1. Concentration-response curves and predicted EC_{50} values of BAP for (A) CYP1A1 and (B) CYP1B1 in normal and IL-13 phenotypes.

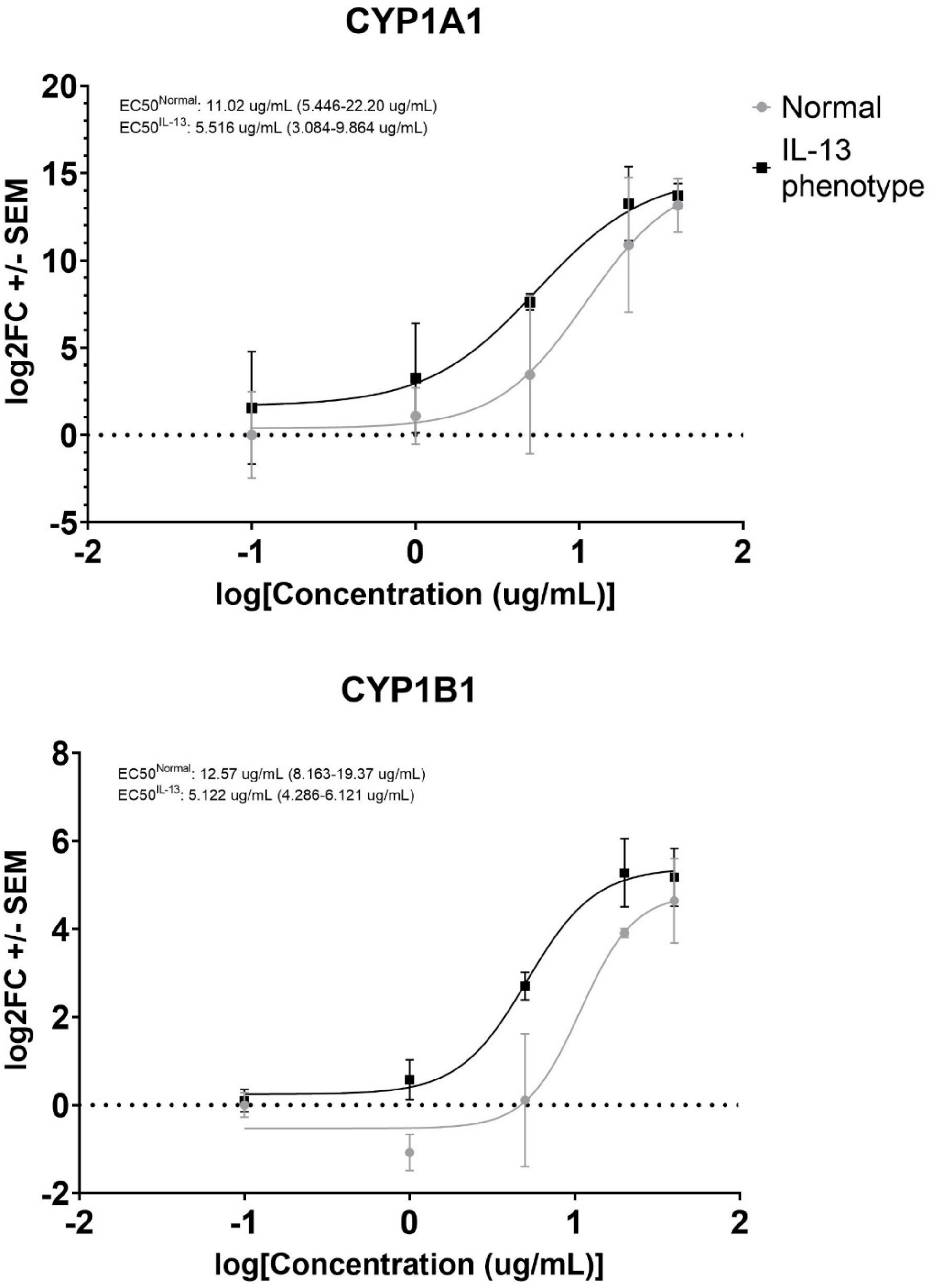

Supplement: Supplementary file 1 [file DataSheet1.PDF]
